# Advances in the REDCAT software package

**DOI:** 10.1186/1471-2105-14-302

**Published:** 2013-10-07

**Authors:** Chris Schmidt, Stephanie J Irausquin, Homayoun Valafar

**Affiliations:** 1 Department of Computer Science & Engineering, University of South Carolina, Columbia, SC 29208, USA

**Keywords:** Protein, Structure, Residual, Dipolar, Coupling, VMD, Xplor-NIH, RDC

## Abstract

**Background:**

Residual Dipolar Couplings (RDCs) have emerged in the past two decades as an informative source of experimental restraints for the study of structure and dynamics of biological macromolecules and complexes. The REDCAT software package was previously introduced for the analysis of molecular structures using RDC data. Here we report additional features that have been included in this software package in order to expand the scope of its analyses. We first discuss the features that enhance REDCATs user-friendly nature, such as the integration of a number of analyses into one single operation and enabling convenient examination of a structural ensemble in order to identify the most suitable structure. We then describe the new features which expand the scope of RDC analyses, performing exercises that utilize both synthetic and experimental data to illustrate and evaluate different features with regard to structure refinement and structure validation.

**Results:**

We establish the seamless interaction that takes place between REDCAT, VMD, and Xplor-NIH in demonstrations that utilize our newly developed REDCAT-VMD and XplorGUI interfaces. These modules enable visualization of RDC analysis results on the molecular structure displayed in VMD and refinement of structures with Xplor-NIH, respectively. We also highlight REDCAT’s Error-Analysis feature in reporting the localized fitness of a structure to RDC data, which provides a more effective means of recognizing local structural anomalies. This allows for structurally sound regions of a molecule to be identified, and for any refinement efforts to be focused solely on locally distorted regions.

**Conclusions:**

The newly engineered REDCAT software package, which is available for download via the WWW from http://ifestos.cse.sc.edu, has been developed in the Object Oriented C++ environment. Our most recent enhancements to REDCAT serve to provide a more complete RDC analysis suite, while also accommodating a more user-friendly experience, and will be of great interest to the community of researchers and developers since it hides the complications of software development.

## Background

Residual Dipolar Couplings (RDCs) have emerged within the last two decades as a powerful source of data that can be acquired by Nuclear Magnetic Resonance (NMR) spectroscopy. RDCs can be used for several purposes, but the primary impetus in their use is the study of structure and dynamics of biomolecules in solution [1]. This is attributed to their ability to provide structural information at atomic resolution, while also containing sensitivity to motions ranging from time scales of picoseconds to milliseconds [2-5]. RDCs have been used in studies of carbohydrates [6-10], nucleic acids [11-16], proteins [17-24] and small molecules [25,26]. Their utility has also been demonstrated in various applications including: investigations of protein backbone structure [23,27,28], development of powerful assignment strategies [29,30], and the simultaneous examination of structure and dynamics of target molecules [31-33]. In summary, RDCs can be used as informative, accurate, and economical probes of structure and internal dynamics for both routine and challenging macromolecules [12,31,34-36].

Historically, the use of RDCs has been limited by two main factors: sample preparation, and data analysis. The introduction of a variety of alignment media [37-39], combined with advances in instrumentation [40] and data acquisition, have mitigated the experimental limitations in obtaining RDCs. The major challenge in utilization of RDC data in recent years has been in disentangling the various components which it encapsulates. This task is particularly challenging considering that an individual RDC datum reports valuable information related to the overall tumbling and preferred orientation of a molecule, as well as the relative orientation of each individual interaction vector within the alignment frame. Therefore the main limiting factor in full utilization of RDC data has been a lack of powerful, and yet user-friendly, RDC analysis tools capable of extracting the pertinent information that is embedded within this complex source of data.

Nearly all of the currently existing NMR data-analysis software packages such as Xplor-NIH [41], CNS [42], CYANA [43], DYANA [44] or MSpin [45] have been modified to include RDC data as additional restraints in their analyses. RDC data have also been incorporated into some popular molecular dynamic simulation packages such as Amber [46] and GROMACS [47]. Despite these adaptations, structural refinement of biological macromolecules from RDC data continues to be a non-trivial task. The proper use of RDC data is further hindered by an iterative process that normally consists of three distinct steps. During the first step, an initial structure is evaluated for fitness to RDC data [48,49]. During the second step, structural refinement software is deployed for refinement of an initial structure that may be several angstroms away from the native structure (as measured over the backbone atoms). Related to this step, various mechanisms have been introduced [48-54] for the estimated order parameters or order tensors to prime the search mechanisms of the refinement tools. Finally, a third step often consists of visual inspection of the refined structure using programs such as Molmol [55], Pymol [56] or VMD [57]. This entire process, of structure refinement from RDC data, may be manually repeated until convergence to an optimal structure. However, a number of pragmatic and theoretical limitations are normally encountered during the refinement of macromolecular structures from RDC data. These limitations include activities such as the conversion of file formats and the transferring of results from one analysis software to another, which are tedious but important. Another category of challenges associated with the study of RDCs is selecting the most optimal mechanism of structure refinement using RDC restraints. Examples include: selection of the most representative order tensor/s during the refinement process, selection of region/s that should be subjected to a refinement procedure, or determining the aggressive nature of a refinement process (temperature scheme of annealing).

Here we report advances in the REDCAT [48] software package, which address several of the aforementioned hindrances in an effort to promote and expedite more effective analyses of RDC data. This latest version of REDCAT incorporates several new features including combined analyses, inclusion of a flexible selection mechanism, importing/exporting functions, improvement of the core computational engine, and the release of its source code under the GNU open-source licensing. In addition, interfaces have been developed that allow for direct interaction of REDCAT with VMD [57] and Xplor-NIH [41]. In this report, we describe each new feature and its utility in detail. We also reveal the results obtained in the testing of these features with respect to structure refinement and validation using computed and experimental RDC data. The latest software package is available for download via the WWW from http://ifestos.cse.sc.edu.

### Theory

Theoretical and experimental aspects of RDCs have been extensively presented in the literature. However, in order to facilitate a more informed discussion, here we include a very brief overview of RDC theory as it relates to the presented work.

Residual Dipolar Couplings (RDCs) are derived from the interaction of two magnetic dipoles, when in the presence of the external magnetic field of an NMR instrument [35,58]. This interaction yields information regarding the average orientation of two nuclei relative to the magnetic field (Equation 1).

(1) $$\mathrm{RD}C^{\mathrm{ij}}=-\left(\frac{\mu_{0}\gamma_{i}\gamma_{j}h}{{\left(2\pi r_{\mathrm{ij}}\right)}^{3}}\right)\cdot \langle \frac{{3\cos}^{2}\theta_{\mathrm{ij}}\left(t\right)-1}{2}\rangle$$

In Equation 1, *RDC*^*ij*^ is the RDC between nuclei *i* and *j*, *μ*_*0*_ is the magnetic permeability of free space, *h* is Planck’s constant, *γ*_*i*_ and *γ*_*j*_ are the nuclear specific gyromagnetic ratios for atoms of type *i* and *j*, *r*_*ij*_ is the distance between nuclei *i* and *j* (in units of Angstrom), and *θ(t)* is the time dependent angle between *B*_*0*_ and the vector adjoining nuclei *i* and *j*. REDCAT utilizes an expanded form of Equation 1, shown in Equation 2, and its vector notation (refer to Equation 3).

(2) $$\begin{array}{l} D_{\mathrm{ij}}=\frac{D_{\max}^{\mathrm{ij}}}{r^{3}}\left[\left(x_{\mathrm{ij}}^{2}-z_{\mathrm{ij}}^{2}\right)s_{\mathrm{xx}}+\left(y_{\mathrm{ij}}^{2}-z_{\mathrm{ij}}^{2}\right)s_{\mathrm{yy}}+z_{\mathrm{ij}}^{2}s_{\mathrm{zz}}\right)\\ \;\left(+2x_{\mathrm{ij}}y_{\mathrm{ij}}s_{\mathrm{xy}}+2x_{\mathrm{ij}}z_{\mathrm{ij}}s_{\mathrm{xz}}+2y_{\mathrm{ij}}z_{\mathrm{ij}}s_{\mathrm{yz}}\right] \end{array}$$

(3) $$D_{\mathrm{ij}}=\frac{D_{\max}^{\mathrm{ij}}}{r^{3}}\bar{v}\cdot S\cdot {\bar{v}}^{T}$$

In Equation 2, $D_{\max}^{\mathrm{ij}}$ is the maximum observable RDC value for a pair of nuclei *i* and *j*, when separated by 1.0Å; *x*, *y* and *z* represent the normalized coordinates of the vector adjoining nuclei *i* and *j*; and *s*_*kl*_ denotes the individual elements of an order tensor matrix. Reformulation of RDCs, as shown in Equation 3, provides a computationally friendlier form of the RDC interaction. In this equation, *S* refers to the *Saupe* order tensor matrix [48,51,59] and *v* represents the normalized interacting vector.

Available RDC data from multiple sites on a protein can be combined into a single linear algebraic representation, shown in Equation 4. This *Ax=b* representation of RDCs enables the use of Singular Value Decomposition (SVD) [48,51,60,61] to easily obtain the optimal order tensor matrix. In Equation 4, the matrix *A* is computed from the coordinates of the interacting vectors, *x* corresponds to the vector representation of an order tensor, and *b* corresponds to the observed values of the RDC data. Furthermore, in this equation, the traceless property of the order tensor is utilized to calculate *S*_*zz*_ from *S*_*xx*_ and *S*_*yy*_, in order to reduce the six variables of the order tensor vector to five. Elimination of the *S*_*zz*_ term is the reason for the appearance of the *z*^*2*^ term in the first two columns of the *A* matrix in Equation 4. Other modifications of the system of equations shown in Equation 4, with their corresponding adaptations of SVD, have also been introduced in order to accommodate conformational rotation of side chain methyl and phenyl groups [62,63].

(4) $$\begin{array}{l} {\left(\begin{array}{lllll} x_{1}^{2}-z_{1}^{2} & y_{1}^{2}-z_{1}^{2} & 2x_{1}y_{1} & 2x_{1}z_{1} & 2y_{1}z_{1}\\ x_{2}^{2}-z_{1}^{2} & y_{2}^{2}-z_{2}^{2} & 2x_{2}y_{2} & 2x_{2}z_{2} & 2y_{2}z_{2}\\ \vdots & \vdots & \vdots & \vdots & \vdots \\ x_{n}^{2}-z_{n}^{2} & y_{n}^{2}-z_{n}^{2} & 2x_{n}y_{n} & 2x_{n}z_{n} & 2y_{n}z_{n} \end{array}\right)\;}_{\left(n\times 5\right)}\cdot {\left(\begin{array}{l} s_{\mathrm{xx}}\\ s_{\mathrm{yy}}\\ s_{\mathrm{xy}}\\ s_{\mathrm{xz}}\\ s_{\mathrm{yz}} \end{array}\right)\;}_{\left(5\times 1\right)}\\ ={\left(\begin{array}{l} D_{1}\cdot \left(r_{1}^{3}/D_{\max}\right)\\ D_{2}\cdot \left(r_{2}^{3}/D_{\max}\right)\\ \vdots \\ D_{n}\cdot \left(r_{n}^{3}/D_{\max}\right) \end{array}\right)\;}_{\left(n\times 1\right)} \end{array}$$

## Implementation

In this section we first begin with a detailed list of REDCAT’s most recent advancements, which illustrate the many improvements in overall usability and functional analyses. This is followed by a disclosure of software engineering strategies incorporated into REDCAT and its VMD-REDCAT and Xplor-GUI modules. Finally we conclude this section with a description of our software testing and validation procedure; the purpose of which is to demonstrate some of REDCAT’s newest features as they pertain to protein structure refinement and validation.

### REDCAT improvements and new features

The REDCAT software package [48] was designed for evaluation of biomolecular structures using RDC data by comparing experimental RDCs to back-computed RDCs. In addition to providing a measure of structural fitness to the experimental data, REDCAT provides various visual means of inspecting the results (such as Sanson-Flamsteed projections) through an interaction with the gnuplot [64] package. The REDCAT software package has been updated to incorporate additional analysis features, integrate more visualization tools and reflect modern software engineering principles. In this section we begin by first introducing some of the features that improve the overall software usability, this is followed by features that improve analysis of RDC data. The former set of features is briefly discussed, with corresponding graphical dialogues depicted in the subsection (“Improvement in overall usability”) that follows. The latter set of features have been evaluated using a number of scenarios and are presented in the “Improvements in functional analyses” subsection of this section, with related results reported and examined in “Results and discussion”. Detailed descriptions of each feature, as well as a user’s guide, are available through user documentation at http://ifestos.cse.sc.edu/REDCAT/documentation and additionally at the following Wiki page http://www.nmr2.buffalo.edu/nesg.wiki/REDCAT.

#### Improvement in overall usability

**Save/Load State –** Based on user requests, we have incorporated a more flexible means of saving and loading REDCAT analysis sessions. The Save and Load State feature allows the user to take a snapshot of all internal variables at any given time. This provides the user with the opportunity to return to the exact same session at a later time, or to share the saved state of an analysis with others for further inquiry and/or collaboration.

**Figure 1:**
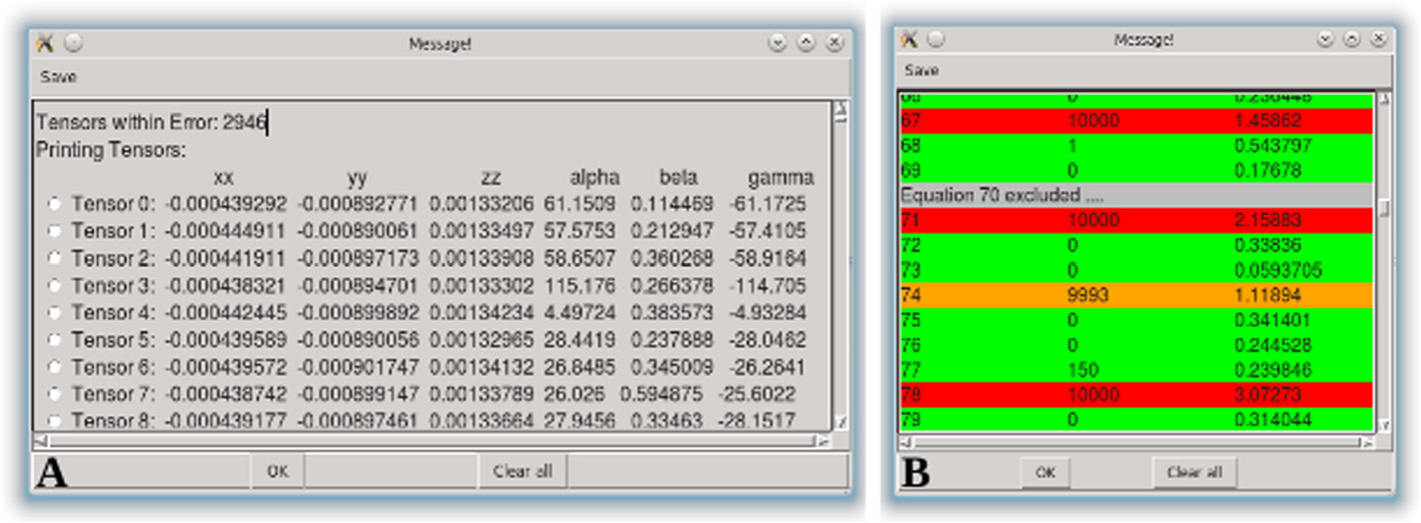
Screen shots of the (A) Get Solutions and (B) Rejection/Error Analysis windows.

**Get Solutions and Rejection/Error Analysis –** In the previous version of REDCAT, attainment of the “Best Solution” (least squares solution) and the collection of all possible solutions were performed in separate steps. Furthermore, analysis of residual errors that were generated by each datum was performed in multiple stages. Proper analysis and interpretation of the final results is better facilitated by integrating all available information. Therefore these analyses have now been combined to provide a single list - with the least squares solution presented as the first entry, followed by any other suboptimal order tensors that satisfy the RDC data to within the indicated errors (shown in Figure 1A). Similarly, statistics related to the Monte Carlo sampling of the solution space and error analysis have been combined into a single error analysis feature (shown in Figure 1B). The radio buttons shown in Figure 1A can be used to select an individual order tensor for use with other functions of REDCAT, such as back-calculation of RDCs.

**Figure 2:**
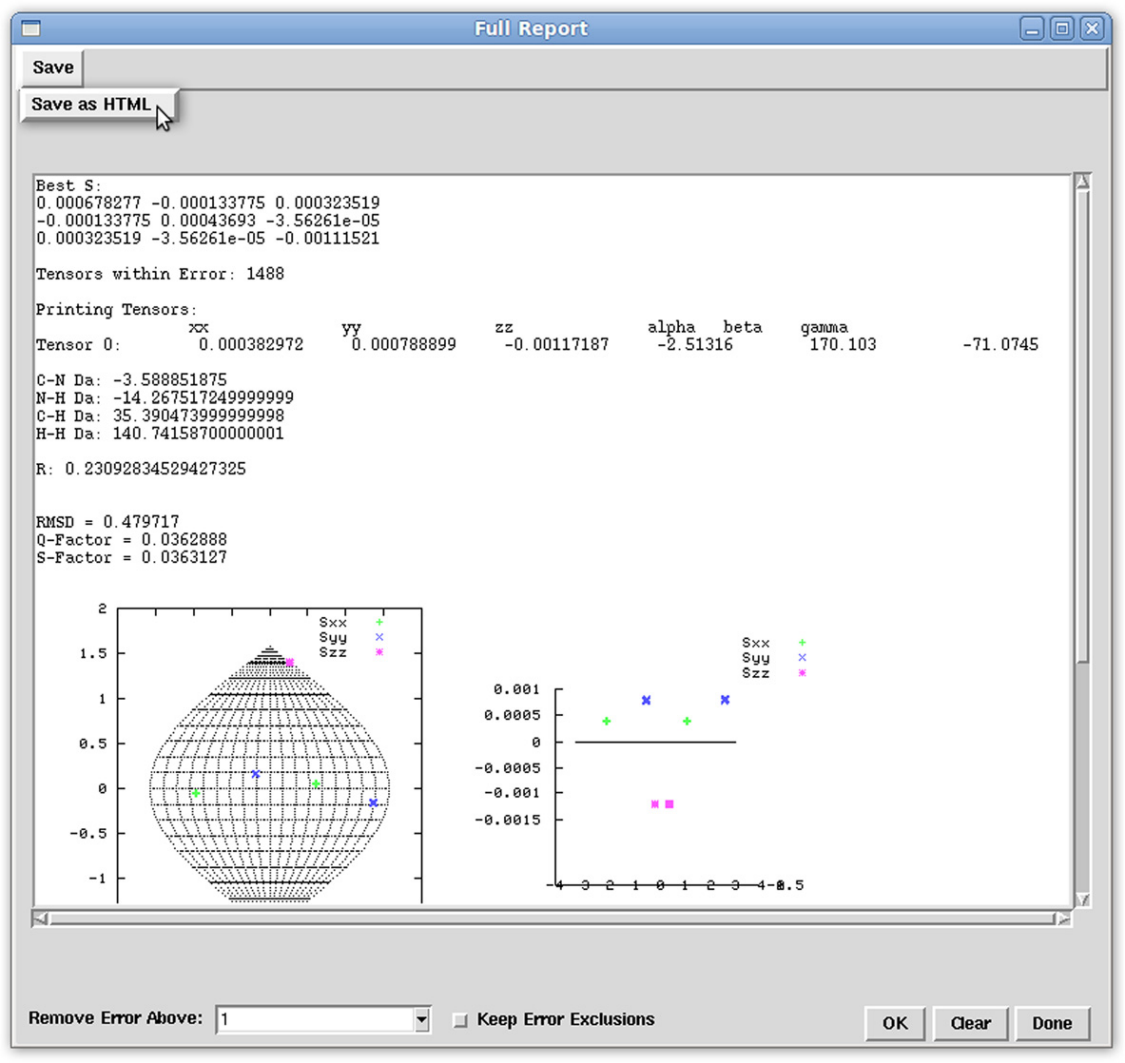
Screen shot of the REDCAT Full Report window.

**Generate Report –** RDC data may be used to scrutinize the fitness of a given structure. Although this task can be accomplished by simply observing the RMSD or Q-factor scores of a given structure to the experimental RDC data, a more thorough analysis requires observing the residual error contribution of each vector as well as the comparison of order tensors and a number of other informative items. In addition, this process may be repeated for every alignment medium in instances where RDC data are collected in several media. This process of collecting the individual pieces of information from various analyses is both cumbersome and time consuming. Therefore, a new feature has been added that combines the results of various analyses into a summary report. This feature allows a user to select error boundary and exclusion criteria, and performs a number of related REDCAT analyses on the loaded data while displaying all the information in one convenient location. The “Full Report” window, partially shown in Figure 2, summarizes the essential information by providing the following: the optimal Saupe order tensor matrix; the number of tensors within error; the decomposed Euler rotation and order parameters of the optimal Saupe order tensor; the rhombic and axial components of the anisotropy (*D*_*a*_ and *R*) for C-N, N-H, C-H, H-H; RDC-RMSD and Q-Factor scores; SF plots showing all possible Monte Carlo tensors within error; a plot of residual errors and the entries that cause violations; a correlation plot comparing back-computed versus experimental RDC data; and a list of all equations omitted from analysis due to violation of errors. Another useful feature is the option of saving this report in HTML format which can then be incorporated into a word processing document, viewed in common web browsers, or shared directly on the web.

**Figure 3:**
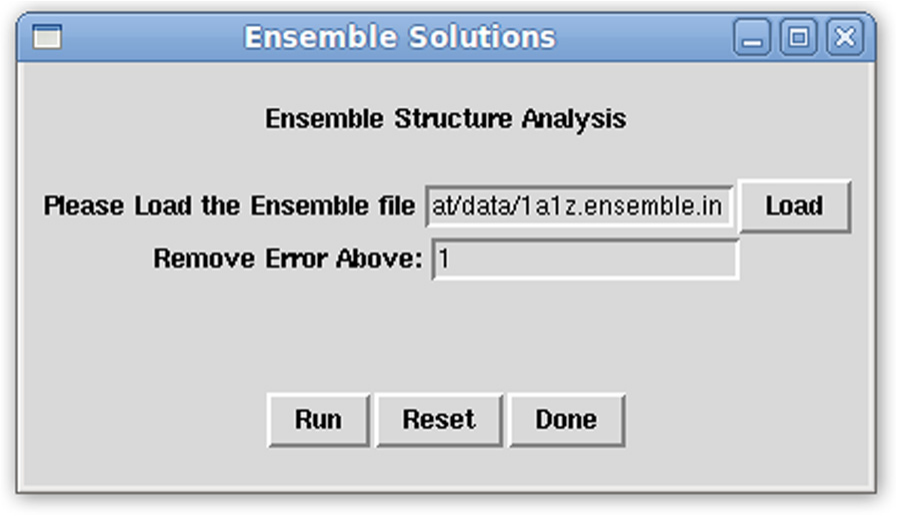
Screen shot of REDCAT Ensemble Solutions window.

**Ensemble Solutions –** RDC data can also be used to select the most suitable structure from an ensemble of available structures. Such analysis sessions require examination of every structure in every alignment medium. To facilitate the collection of all relevant information for every structure in every alignment medium, the “Ensemble Solutions” feature (shown in Figure 3) has been implemented in REDCAT. This tool can perform automated analysis on multiple REDCAT files and display a complete report in one convenient HTML file. An ensemble file, which contains a list of absolute paths to the prepared REDCAT files, is loaded and the maximum tolerable error is specified in units of Hz. The “Run” button will perform the analysis and present the integrated results in the Message window. The “Reset” button clears the two entry boxes in the Ensemble Solutions window, and the “Done” button hides the window. Upon execution of the Ensemble Analysis, the user is presented with the same information presented in the Full Report window (shown in Figure 2) for each structure. By using several REDCAT files for the same protein structure, but different alignment media, users are able to quickly compare the fitness of a protein structure to all collected RDC data. In addition, multiple refinements of a single structure can be compared with the same RDC data in order to find the protein structure that best conforms to the data.

**Figure 4:**
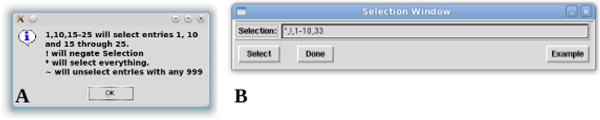
Screen shot of (A) help dialogue for the selection feature of REDCAT and (B) the REDCAT Selection window with an example selection statement.

**Selection –** The Selection feature enables users to include or exclude various entries, or blocks of entries, in the RDC analysis by creating a selection statement based on a predefined syntax. The list of predefined selection commands can be obtained through the help dialogue of this command (shown in Figure 4A): the “*” character is used to select all, “!” is used to negate the current selection, “~” is used to exclude any incomplete data sets (any entry with a 999), “-” is used to indicate a range of entries starting with the first number and ending with the second number (inclusively), and “,” is used to separate individual commands. Using the selection illustrated in Figure 4B as an example, the command “*,!,1-10,33” first selects all equations, negates the selection (thereby deselecting all equations), and then selects equations 1 through 10 and 33.

**Figure 5:**
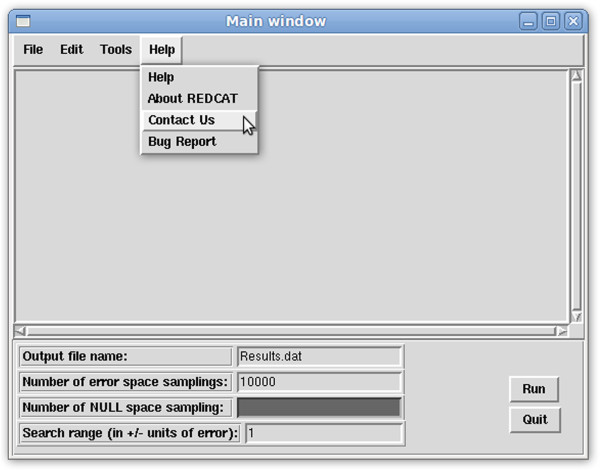
Screen shot of REDCAT’s Help feature.

**Interaction with the User Community and Feedback –** A help feature has also been included, which opens the user’s default browser to the on-line REDCAT manual. This manual helps to describe the various features and menus of REDCAT, and gives several helpful tutorials to aid users in familiarizing themselves with the software. A “Contact Us” feature, highlighted in Figure 5, has also been added to open the user’s default mail client and send a message to a Gmail account registered to Dr. Valafar’s laboratory (valafarlab@gmail.com).

#### Improvements in functional analyses

**Figure 6:**
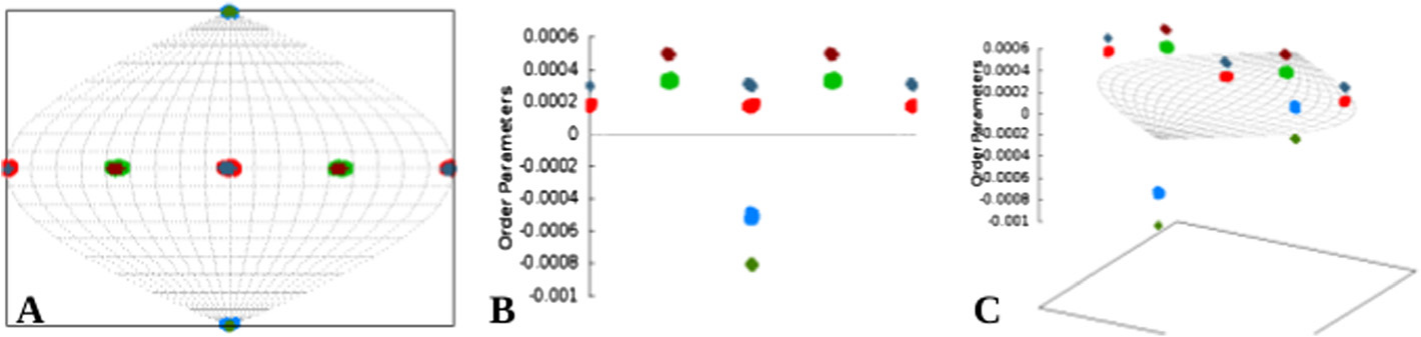
**A 3D-SF plot used in assembly of the dynamical terminal helix of 1A1Z.** Panel **(A)** corresponds to the top view of the 3D-SF plot that is identical to a 2D-SF plot. Panel **(B)** corresponds to the side view of the 3D-SF plot where the z-axis consists of the magnitudes of order parameters. Panel **(C)** is an intermediate view of the 3D-SF plot as it rotates from top-view to side-view.

**3D-SF plot –** Another advantage afforded by RDC data is the ability to assemble fragments and molecular complexes [20,65,66]. During such exercises, the structure of individual components within a larger complex are determined individually; this is followed by the assembly of all individual fragments into a final complex oriented by using RDC data. This is typically accomplished through the reorientation of each element of a complex, such that the orientational components of the order tensors from each domain overlap as illustrated in Figure 6A. However, the underlying principle that enables such an assembly process is the assumption of the rigidity of the individual members of a larger complex with respect to each other (therefore they share the same order parameters [35,67]). Violation of this fundamental assumption can produce erroneous assembly of the individual domains. Traditionally, two-dimensional SF plots (2D-SF) have been used for visualization of the orientational components of the anisotropy. While a 2D-SF plot provides the means to examine the orientational components of the anisotropy, it fails to provide any mechanism of confirming the rigidity assumption. A 3D-SF plot has been included within REDCAT to facilitate a complete comparison of order tensors by including the magnitude of individual order parameters along the z-axis. Therefore a top view of the 3D-SF plot will produce the traditional 2D-SF plot, while a side-view of the plot will provide the comparison of the individual order parameters. More complete examples of this feature are included in the “3D-SF Plot” section of “Results and discussion”.

**VMD Extension –** Visual Molecular Dynamics (VMD) [57] is a molecular visualization program that also serves as a front end for other protein analysis tools such as NAMD [68]. VMD allows users to visualize and examine a protein structure in many different ways. This can prove useful for finding incorrect structural regions, and allows for a more focused refinement. VMD also has a Tool Control Language (TCL) interface, wherein a user may send TCL commands via a script or line-by-line to VMD in order to access and modify data associated with the loaded molecular model. Through the TCL interface, it is possible to create extensions that can be registered with VMD in order to take advantage of VMD’s capabilities. Some desirable features include: parsing many atomic coordinate file formats, the ability to create three-dimensional annotations on an existing molecular model, properly orienting the molecular model, and saving a molecular model’s atomic coordinates in numerous file formats. Using this communication mechanism, VMD-REDCAT has been developed in order to take advantage of these features. As an extension of VMD, VMD-REDCAT is designed to include all features of the stand-alone REDCAT, while also including new features that allow the user to visualize the resulting RDC analyses on the modeled molecular structures. Such an extension provides a number of advantages in devising better strategies for analysis of RDC data. For example, violation of structural fitness to the RDC data can be easily justified when the violations occur in regions that contain a loop or are unstructured; this is not the case for violations internal to secondary structural elements. Therefore the resulting color display of different interacting vectors can help to appropriately place the violations within a structure. Furthermore, due to the nonlinear nature of the RDC interaction, and depending on the orientation of a vector, a large violation may translate into a slight or large reorientation of the vector in order for that vector to satisfy its RDC constraint. It is therefore useful to display all possible orientations that correspond to a given RDC datum and its acceptable error. The VMD-REDCAT extension provides an interactive mechanism of facilitating both of the above visualizations. Various examples of these two features are illustrated in the “Visualization of RDC analyses with VMD-REDCAT” section of “Results and discussion”.

**Interface to Xplor-NIH –** Xplor-NIH [41,69] has evolved over the years to become a powerful software suite that allows users to utilize different experimental datasets including: NOE, RDC, Dihedral, Ramachandran, and standard atomic geometric constraints. The combination of constraints with common scripting techniques, such as looping and setting variables, enables the implementation of complex annealing simulations and minimization routines. Programs such as Xplor-NIH play an important role in protein structure calculation and refinement from experimental data using their internal molecular mechanics or molecular dynamics simulation engines.

**Figure 7:**
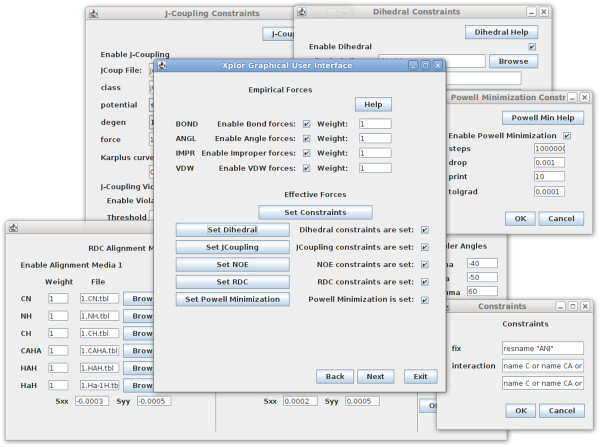
Screen shot depicting several dialogues of the XplorGUI interface.

Currently, the common practice in utilization of Xplor-NIH is to keep template scripts that perform routine tasks and simply change the variables – making modifications to these scripts to customize the refinement of individual proteins. Specific to the analysis of orientational restraints, the Xplor-NIH scripts are often modified to incorporate information regarding the axial/rhombic components of anisotropy for different types of RDC restraints, inclusion of the alignment frames in the form of virtual atoms, and a number of other steps to complete the analysis requisites. Information related to the alignment tensors can be obtained from programs such as REDCAT [48], PALES [49], nD-RDC [52-54], or maximum likelihood fit of powder patterns [50]. The information obtained from these programs is then manually converted to a format that is compatible with Xplor-NIH. Therefore, the traditional approach of modifying an existing template is cumbersome and can lead to errors with potentially unwarranted outcomes. A more beneficial solution is to utilize a graphical user interface (GUI) to generate template scripts. Interaction with Xplor-NIH through a GUI helps to minimize errors and omissions, since the user can either select options from a provided list of variables and constraints, or choose to set them to default values. In this way, common scripts can be generated pseudo-automatically. This provides the user with a more complete understanding of the script being generated and relieves them from the burden of remembering to modify, add, and/or remove lines in a template script. Providing these beneficial features has motivated the creation of an interaction between REDCAT and Xplor-NIH, which we refer to as XplorGUI. XplorGUI can be launched from within REDCAT and easily facilitates refinement of protein structures by Xplor-NIH. This interaction allows for easy and seamless transfer of the needed information from REDCAT to XplorGUI. A collection of the interacting windows of XplorGUI are shown in Figure 7.

The interface operates based on a Wizard Style, with categories pertaining to Xplor-NIH’s Internal Variable Model (IVM). Individual windows relate to empirical forces, NOE, Dihedral, J-Coupling, RDC, and Powell Minimization have been created to aid the user in utilizing and incorporating these constraints. All force terms can be easily turned on and off, and help buttons that provide the user with links to relevant portions of Xplor-NIH’s on-line manual are included [70]. Once the Xplor-NIH parameters are properly configured, the corresponding Xplor-NIH script is created and executed while saving the output. Scripts generated in this manner are useful for common tasks and can be expanded to perform more detailed procedures. The wizard style XplorGUI also presents users with an opportunity to insert a preexisting script within the automatically created one, thereby facilitating a more flexible inclusion of customized protocols. Examples demonstrating the utility of this feature are discussed in the “Structure refinement with XplorGUI” section of “Results and discussion”.

### Software engineering

The REDCAT software package is composed of four distinct parts: the C++ REDCAT computational engine, the REDCAT front-end interface (REDCAT.tcl), an interface between VMD and REDCAT (VMD-REDCAT), and an interface between Xplor-NIH and REDCAT (XplorGUI). The software specifications of each are presented in the following subsections.

#### C++ REDCAT computational engine

**Figure 8:**
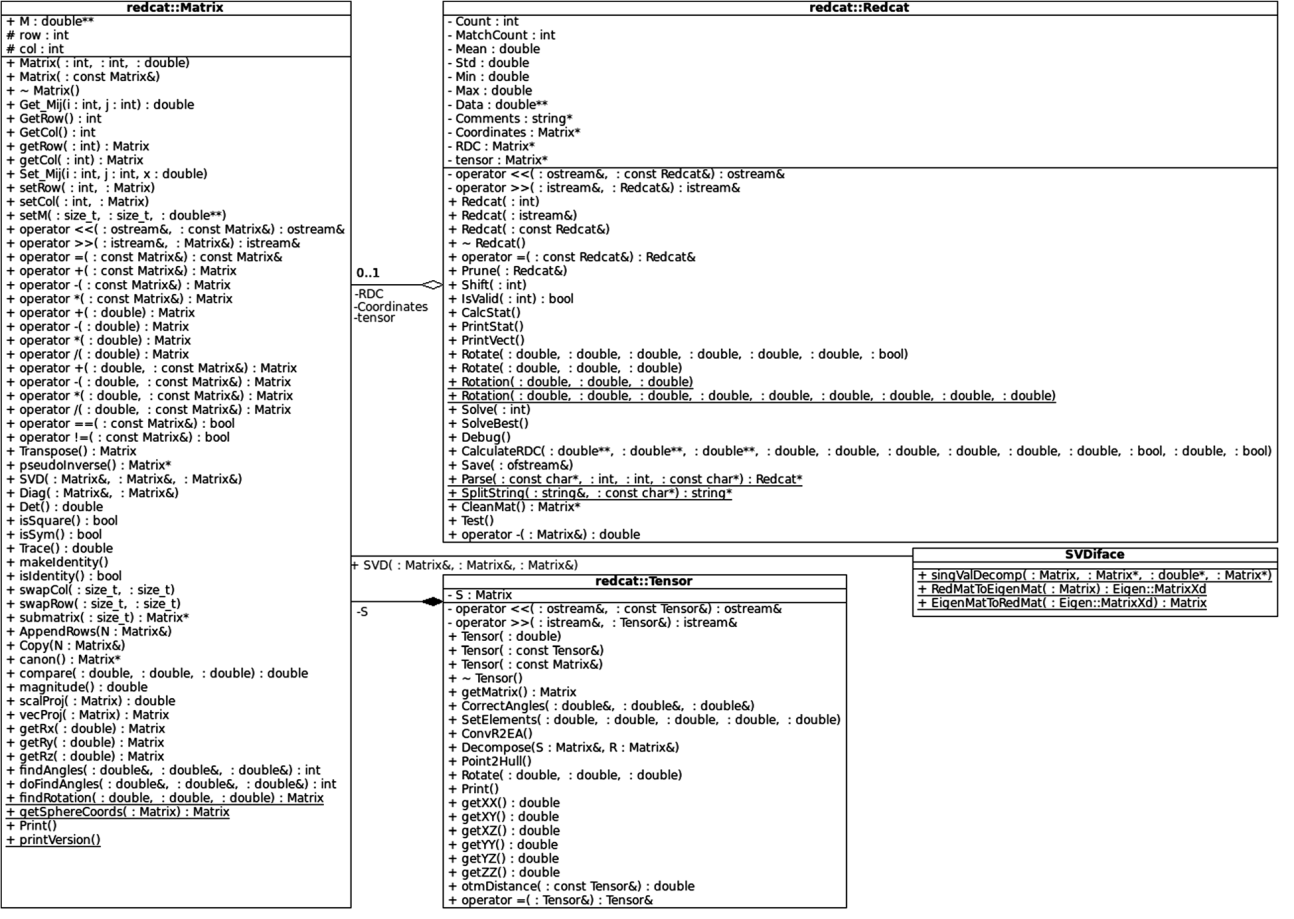
UML class diagram of REDCAT’s C++ computational engine.

The computational engine of the previous version of REDCAT consisted of a collection of programs written in C, Perl, bash, and Tcl/Tk languages. This diverse set of languages, consisting of numerous files, significantly increased the time and effort required in further development of the entire package. To reduce this inefficiency, the overall function of the individual programs was separated into front-end and back-end components of the new REDCAT software model. The computational back-end of the software package has been condensed into an Object Oriented Design paradigm that is more maintainable, easier to read and understand, and more powerful from a scripting stand point. The separate computational engine, combined with its command line flags, allow for direct interaction with REDCAT from a command line terminal in order to create faster and more scalable RDC batch analyses. Figure 8 illustrates the UML diagram of REDCAT’s C++ computational engine. As indicated in Figure 8, SVDiface facilitates intercommunication between REDCAT and the *Eigen3* software package (http://eigen.tuxfamily.org) for Singular Value Decomposition (SVD) [48,61] and other linear algebraic operations. Although the *Eigen3* package is capable of defining and utilizing *Matrix* objects, we have developed our own *Matrix* and *Tensor* objects in order to better customize them for our specific needs. These two objects facilitate handling of their corresponding data objects as shown in Equation 4. The *Redcat* object utilizes *Matrix* to maintain its molecular coordinates as defined previously [48].

#### REDCAT Tcl/Tk front-end interface

The front-end interface to the REDCAT software package has been implemented in the Tcl/Tk scripting language. In addition to maintaining the look-and-feel of the previous version, the Tcl/Tk language offers a simple and modular implementation, is available on many platforms, and interfaces to VMD and Xplor-NIH. REDCAT’s Graphical User Interface (GUI) utilizes wish (a Tcl/Tk interpreter) to handle all data parsing, manipulation of Input/Output, interactions with the back-end computational engine, communication with VMD and Xplor-NIH, and generation of visual elements. All plotting tasks utilize the gnuplot software package (http://www.gnuplot.info/), while creation and conversion of images is performed with use of the ImageMagick software package (http://www.imagemagick.org/script/index.php). Both of these software packages are open-source and available on a number of operating systems including Windows, Linux and Mac OS X.

#### VMD-REDCAT interface

**Figure 9:**
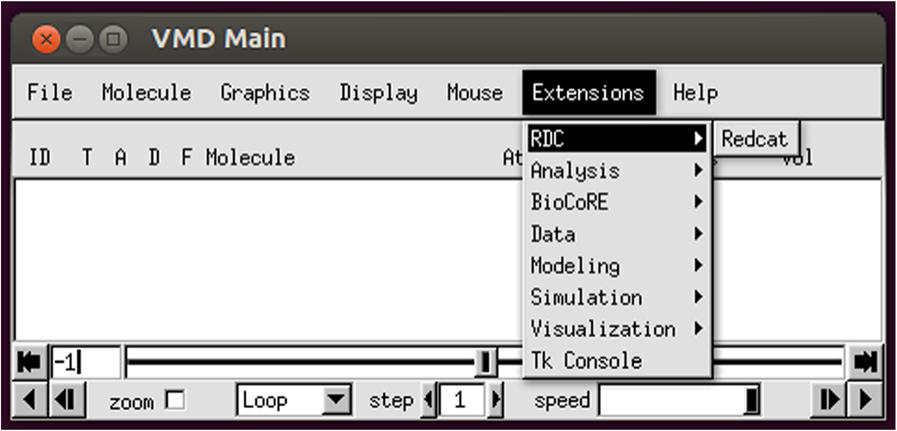
Screen shot illustrating how to access the REDCAT extension in VMD.

The latest version of the REDCAT software package is distributed with an alternative front-end GUI to the REDCAT computational engine, as an extension of the VMD software package. As mentioned previously, this extension is named VMD-REDCAT and it replicates REDCAT.tcl in every way, facilitating a bi-directional communication between the two software packages. VMD-REDCAT takes advantage of the Tcl/Tk extension framework presented in VMD [57,71] and is very similar to the stand-alone REDCAT.tcl version in terms of its software engineering principles. Instructions on how to access VMD-REDCAT are included in the INSTALL-VMDREDCAT readme file, which is located in the vmd subdirectory of Redcat. If the extension is installed correctly, REDCAT may be accessed from VMD. This is illustrated in Figure 9, where Redcat is the first entry on the menu bar under Extensions → RDC.

#### XplorGUI interface

**Figure 10:**
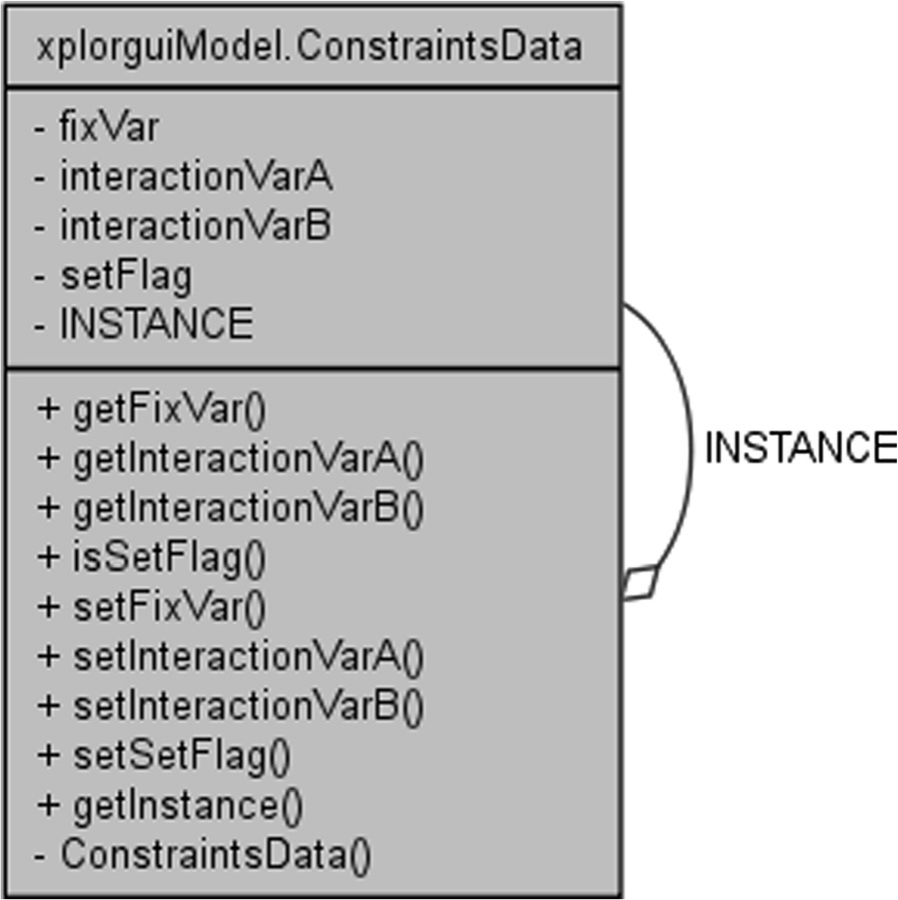
UML class diagram of a representative class from the Model portion of XplorGUI.

**Figure 11:**
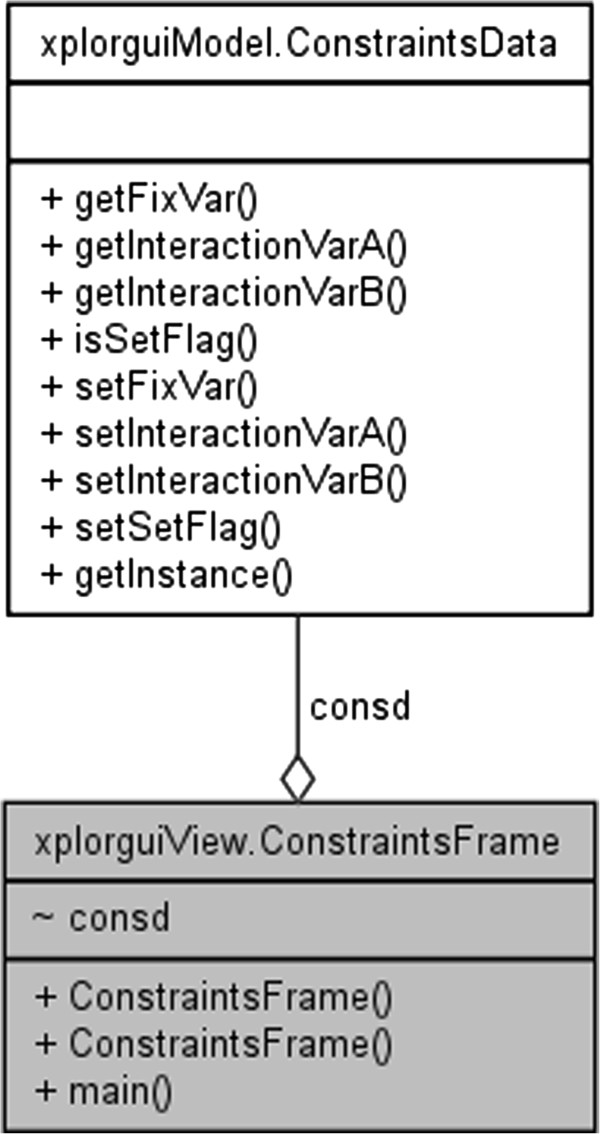
UML class diagram of a representative interaction between the view and model.

**Figure 12:**
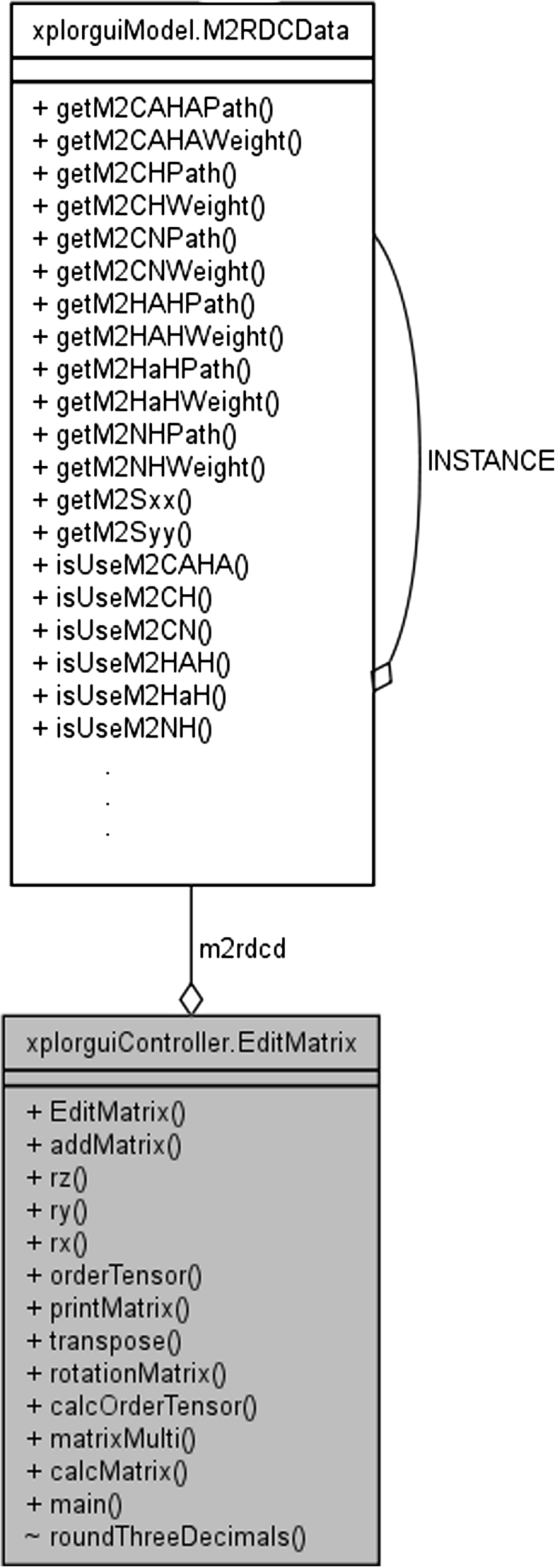
UML class diagram of a representative interaction between the model and controller.

**Figure 13:**
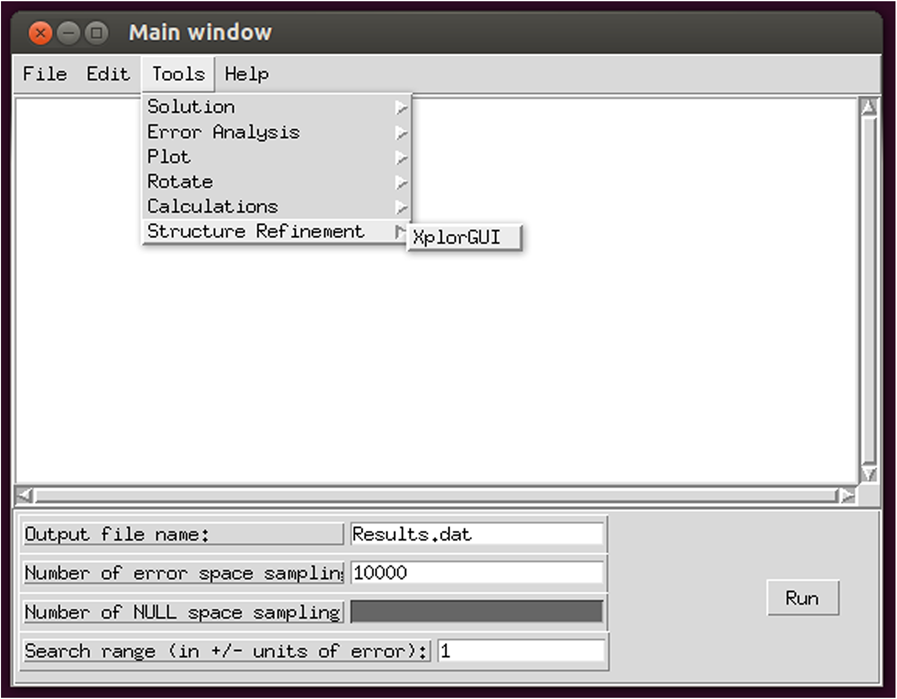
Screen shot depicting how to access the XplorGUI interface from within REDCAT.

The front-end interface to the Xplor-NIH program is designated XplorGUI and is engineered as a Java Swing application utilizing the Object Oriented Design paradigm and a Model-View-Controller design pattern. A UML class diagram of a representative object from the *Model* portion of the XplorGUI is shown in Figure 10. The internal variables of Xplor-NIH are represented in the Model classes (a representative of which is shown in Figure 11), View represents the layer presented to the user (interface layer). The input from the user is communicated to the Model classes for storage. Finally, the main View class (shown in Figure 12) invokes various Controller classes based on the user interaction to create and execute an appropriate script. In this way, the application itself is easily maintained and modular, allowing for inclusion of additional Xplor-NIH restraints as its internal variable model evolves. This accommodates easy modification of the script generation portion of the program by decoupling it from the interface and model, so that as better techniques become available, the appropriate sections can be substituted or modified to take advantage of these improved methods. Once VMD-REDCAT has been launched (refer to section VMD-REDCAT interface), the XplorGUI interface may be accessed through REDCAT as depicted in Figure 13, where XplorGUI is the first entry on the menu bar under Tools → Structure Refinement.

### Software testing and validation

Our software testing and validation efforts consisted of several hierarchical layers. The core of our testing procedure consisted of comparing results of REDCAT’s SVD calculations with that of MATLAB’s to within machine-precision. Additional tests were performed to ensure proper parsing and handling of data by the front-end of REDCAT. In this section we outline the mechanisms which demonstrate the utility of REDCAT’s newest features as they pertain to functional analyses of RDCs. We also highlight the added convenience of linking REDCAT to both VMD and Xplor-NIH, where appropriate.

The general overview of our functional testing strategy consists of utilizing computationally generated data from structures obtained from the Protein Data Bank (PDB) [72] and in some cases by using experimental data obtained from the Biological Magnetic Resonance Bank (BMRB) [73,74]. The use of theoretical data helps to establish both the correct function of REDCAT’s features, and the consistent notation of data transfer to VMD and Xplor-NIH. The application of experimental data is beneficial in illustrating the utility of REDCAT’s features under pragmatic conditions. Details, with regard to each experiment, are described in the following subsections.

#### 3D-SF plot

The most common misuse of the 2D-SF plot is within the context of structure determination of dynamical proteins. Therefore to illustrate the importance of this feature, we resort to a computational model of internal dynamics for the protein PDB:1A1Z. Here the C-terminal helix of the protein (residues 70-83) is subjected to a two-state jump model of motion that was simulated by altering the φ backbone dihedral angle at residue 70 by approximately 60°. RDC data have been generated for both domains of the protein using the same order tensors as shown in Table 1. The observable RDC data for the dynamical domain of this protein has been calculated by averaging the RDC data in both states of dynamics. Because of the existence of internal dynamics, the two domains of this protein (residues 1-69 and residues 70-80) experience different degrees of alignment resulting in two different order tensors. Whereas the assembly of the two domains based on traditional approaches [66] is inapplicable and should be avoided, visualization and scrutiny of the results via Redcat’s 3D-SF plot will help prevent false use of RDCs.

**Table 1:** Order Tensors utilized for generation of synthetic RDC data from the 1A1Z structure

**Medium**	**S**_**xx**_	**S**_**yy**_	**S**_**zz**_	**αα**	**ββ**	**γγ**
M1	−3×10^-4^	−5×10^-4^	8×10^-4^	0º	0º	0º
M2	2×10^-4^	5×10^-4^	−7×10^-4^	−40º	−50º	60º

#### Structure refinement with XplorGUI

Structure refinement using RDC data is a challenging task, it is for this reason that a generally accepted approach by the community of researchers is lacking. Our development of XplorGUI has therefore been focused on providing a flexible mechanism that allows importing of any existing refinement routine, in order to simplify configuration of the parameters that accompany analysis of RDCs in Xplor-NIH. Our general approach in establishing the proper interaction between Xplor-NIH and REDCAT is to deploy a simple Powell minimization as our generic refinement procedure. We will confirm the proper interaction in application to synthetic and experimental RDCs acquired from PDB:1A1Z and PDB:1P7E proteins, respectively.

During the refinement exercises we have utilized synthetic RDC data generated from the structure PDB:1A1Z [75]. Two sets of RDC data were generated in two alignment media using the order tensors shown in Table 1. The magnitudes of these two order tensors correspond to the typically observed axial and rhombic components of anisotropy from experimentally acquired data for proteins of similar size. RDC data were generated and used for the vector set {C^i-1^-N^i^, N-H, C^i-1^-H^i^, C_α_-H_α_, H_α_-H, H_α_^i-1^-H} (six RDC data points per residue in each alignment medium).

Our experiments also included experimental RDC data for the protein structure PDB:1P7E [76], which were obtained from the BMRB database [73,74]. Although RDC data were available from five alignment media, and for a variety of interacting nuclei, we only utilized RDC data from the Bicelle and PEG alignment media for the RDC vector set {N-H, C^i-1^-N, C^i-1^-C_α_, C_α_-H_α_}.

The function of REDCAT, and its interaction with VMD and Xplor-NIH, was first demonstrated in application to a perturbed 1A1Z structure. This was followed by subjecting the perturbed structure to a simplistic refinement process that is included in our Xplor-NIH GUI. Synthetic data without any additional error (0Hz error) was used to validate the minimization by observing an RDC-RMSD that converges to zero, and a refined structure that converges to the known structure. This same process was applied to the 1P7E protein, for which we were able to utilize the available experimentally acquired data.

The original structures of 1A1Z and 1P7E were perturbed in two ways to provide a starting point for evaluation of structures and the refinement process. The first structural perturbation consisted of altering a single phi(φ) backbone torsion angle at residues 41 (by 20°) and 28 (by 25°) of proteins 1A1Z and 1P7E, respectively. The second perturbation consisted of subjecting each of the starting structures to a moderate molecular dynamics simulation at room temperature using NAMD [68]. The altered structures are denoted by the PDBID followed by an extension of .PHI or .NAMD, to reflect the method by which the structural alterations were produced.

#### Structure validation

RDCs can play a critical role in refinement of biomolecular structures. Although indispensable, RDCs do present a number of unforeseen challenges that hinder their potential use. One such example arises when different regions of an initial protein structure (or any biomolecular structure) exhibit varying degrees of agreement with the actual structure. In such instances, traditional approaches to structure refinement are able to reduce the structural violations of the discrepant regions, but at the cost of altering the accurate regions of the structure. This tradeoff for improvement in structural quality is primarily due to the fact that the common order tensor that is used for structure refinement is influenced by the entire protein, therefore any existing error is equally distributed throughout the entire protein. A more efficient method would attempt to isolate and preserve the accurate regions of a structure, thereby limiting structure refinement procedures to only the discrepant regions. Such an approach would allow the most precise estimate of order tensors to be obtained based on the most accurate regions of the protein. This objective can be accomplished through the use of REDCAT’s Error-Analysis feature. This feature has been described previously [48] however it was demonstrated in application to perturbation of a single RDC data point. Here we perform a molecular dynamics simulation, utilizing the human Thymidylate Synthase protein⁠⁠, to demonstrate the success of this REDCAT feature in identifying regions that are in disagreement with the RDC data.

Thymidylate Synthase (TS) is a dimer of two identical subunits required for DNA synthesis in a number of organisms [77,78]. Human Thymidylate Synthase (hTS) differs from that of bacterial TS’s in a number of ways. The ability of its active site loop (residues 181 to 197) to exist in two conformations is one notable difference. Yet another is related to the eukaryotic insert loop (residues 107 to 128) that displays either a well-defined conformation or multiple conformations, depending on the conformation of the active site loop [79-82]. To generate RDC data that reflect internal dynamics, a simulation utilizing chain A of the PDB:1HVY structure [81] was created by fixing the entire protein, with the exception of the eukaryotic insert and active site loop regions and 7 residues before and after each region (100 to 135 and 174 to 204, respectively). The simulation was conducted at 298 K using the Xplor-NIH software package (version 2.28) [41,69]. The resulting 100 structures embedded within the trajectory were visualized in VMD and individual frames were used to compute RDC data for the {N-H, C_α_-H_α_} vector set in two alignment media, utilizing the order tensors listed in Table 1. For each residue and vector type, an average was taken across all trajectory files followed by inclusion of ±1 Hz of uniformly distributed noise. This average value represents the RDC that would be observed by NMR under the assumed model of motion and alignments. This elaborate test case is used to not only demonstrate the ability of REDCAT in quantifying structural fitness to RDC data, but also to demonstrate the possibility of isolating discrepant regions. Identification of regions of poor fitness is very useful in harvesting the correct structural regions, and limiting the extent of structural refinement only to regions with high RDC violations.

## Results and discussion

Here we examine the newest features of REDCAT with regard to functional analyses of RDCs: 3D-SF plot, structure refinement with XplorGUI, and structure validation (each of which was described in the previous section within the context of an experiment). In addition to these individual exercises, we have dedicated the first section for visualization of RDC analyses with VMD-REDCAT, since it recurrently appears during every aspect of RDC analysis.

### Visualization of RDC analyses with VMD-REDCAT

**Figure 14:**
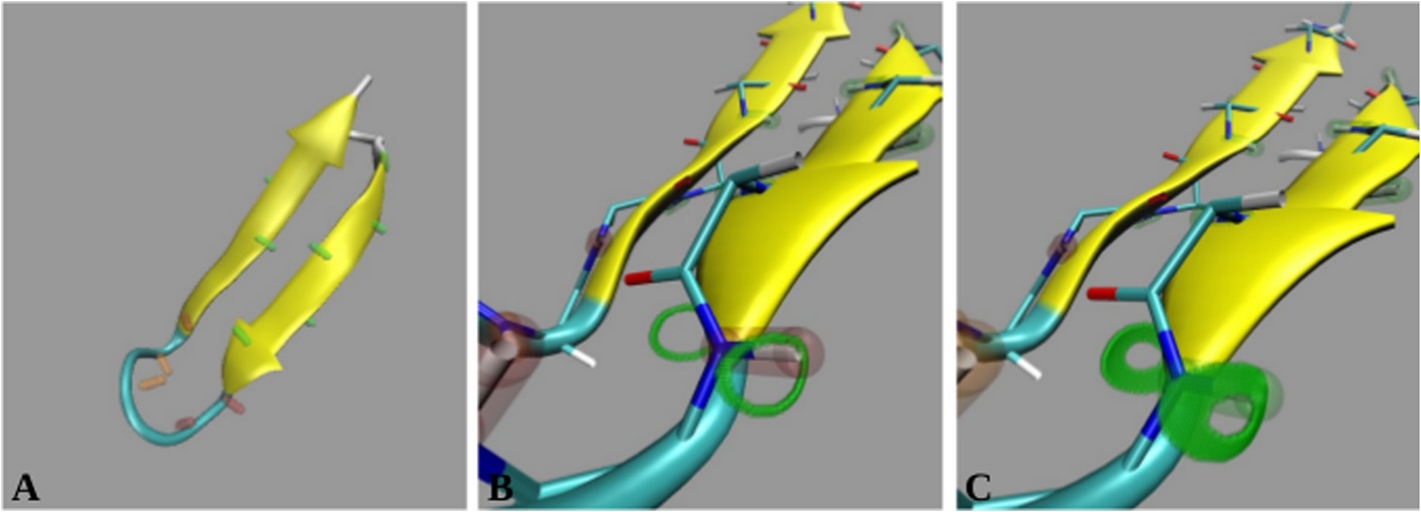
**Visual inspection of the RDC violations for a fragment of the protein 1P7E that is computed by REDCRAFT.** RDC violations for the entire fragment are illustrated in panel **(A)**. Panels **(B)** and **(C)** illustrate the possible orientations of the N-H vector of residue 54 based on error margins of ± 0.5Hz and ±1.5 Hz, respectively.

Conventional approaches to refinement of protein structures from RDC data start by obtaining an order tensor for each alignment medium using an initial structure. Refinement of the initial structure proceeds using programs such as Xplor-NIH, CNS, or CYANA and the estimated order tensors (one for each alignment medium). Although this approach is straight forward to implement, the results may be compromised if either the initial structure or the estimates of the order tensors are poor. A more robust approach to structure refinement can be devised by obtaining an order tensor from more reliable regions of the protein, such as secondary structural elements, that contain acceptable fitness (within the experimental error) to the RDC data. During this process RDC data from the loop or unstructured regions of the protein may be excluded, and is justified based on the potential existence of internal dynamics in these regions. Once a more accurate descriptor of alignment is obtained, the refinement of structure may proceed in application to regions with poor fitness to the RDC data. The VMD-REDCAT interaction easily facilitates such an extensive refinement procedure by way of visualization. An example is provided in Figure 14, where we utilize a fragment of the protein 1P7E (residues 35-56) that has been determined by the software package REDCRAFT [28,33,83] using backbone N-H and C_α_-H_α_ RDC data. Figure 14A illustrates REDCAT-VMD analysis of the structural fitness of this fragment to the RDC data. In this figure the interacting vectors displayed in green, orange and red correspond to vectors that fully satisfy the RDC data, partially satisfy the RDC data, and violate the RDC restraints, respectively. While there are clear violations of the RDC restraints, the visualization of these violations provides the added benefit of interpreting the RDC fitness within the context of the structure. The appearance of RDC violations in the loop region of this fragment therefore mitigates their impact.

Another advantage of visual inspection using REDCAT-VMD is to gain a geometrical perspective of the RDC violations. Due to the highly nonlinear nature of the RDC interaction, violation of RDC data by a certain margin may translate to either a very small or quite large orientational violation. REDCAT-VMD provides the means for this inspection by displaying the acceptable regions of space where a given vector can be oriented. The green bands in Figure 14B illustrate the acceptable orientations, which are within ± 0.5 Hz of error, of the N-H vector of residue 54. This vector is illustrated in red based on its residual RDC error of ±1.5 Hz that exceeds the allotted ± 0.5 Hz of experimental error. It is clear from this figure that although the N-H vector is in violation, its current orientation is very close to the acceptable region. An increase in the amount of tolerable error (to ±1.5 Hz) for this N-H vector expands the plausible orientations, as illustrated by the green bands in Figure 14C, and since its orientation is found to be within the acceptable regions it is now displayed in green.

### 3D-SF plot

The functionality of a 3D-SF plot is instrumental during the assembly of molecular domains by RDC data. This analysis provides all of the information reported by its 2D predecessor, with the added benefit of providing information related to the magnitude of alignment. Here we illustrate the importance of the 3D-SF plot in application to the simulated dynamics of PDB:1A1Z. Our approach consisted of orienting the two domains of this protein (residues 1-69 and 70-83) with respect to each other as described previously [20,66], utilizing the resulting 2D-SF plot illustrated in Figure 6A. In the absence of information related to the magnitude of alignments, this figure would falsely confirm the correct assembly of the two domains since the order tensors from both domains overlap. However, side-view of the 3D-SF plot clearly illustrates the differences in the two order tensors (refer to Figure 6B) as they are separated by the magnitude of their respective order parameters. Figure 6C provides an intermediate view of the 3D-SF plot as it is rotated from top-view to side-view. A successful alignment of the two domains should result in overlap of order tensors from all perspectives of the 3D-SF plot and in all alignment media. In summary, the inclusion of a third dimension within the SF plot provides a more complete visualization tool by including the magnitude of alignment as well as the orientation component of anisotropy.

### Structure refinement with XplorGUI

Our examination of the REDCAT-XplorGUI interaction starts by utilizing the computationally generated and ideal (0Hz error) RDCs from the protein 1A1Z in two alignment media. Although the use of computed and error-free may not fully reflect the challenges in analysis of RDC data, it serves a critical function for debugging purposes since the exact correct answer is always known. The first step in our examination process consisted of evaluating and refining the actual structure of 1A1Z without any structural alteration. Theoretically, the outcome of this exercise should consist of structural fitness to the RDC data near 0Hz, and the refined structure should exhibit a structure that is nearly identical to the starting structure (as indicated by a bb-rmsd value near 0Å). Any outcome other than the theoretically expected one may be attributed to an improper use of the RDC restraints, incompatible notations in transfer of order tensors between REDCAT and XplorGUI, or improper use of the Powell minimization. The first entry in Table 2 lists the results of this exercise and the outcome closely agree with the theoretically expected results. Any subtle discrepancies may be attributed to differences between the actual structure and the utilized force field (for example N-H bond length). The second exercise tests the REDCAT/Xplor-GUI interaction more thoroughly by refining a structure of 1A1Z that has been altered by modification of a single torsion angle. Here, the starting structure exhibits approximately 2.3Hz of structural fitness to the RDC data, while the refined structure exhibits a clearly improved fitness to the RDC data (~0.15Hz) as shown in the second entry of Table 2. This level of improvement based on the RDC restraints corresponds to an optimal improvement of the structure from 1.99Å to nearly 0Å, when measured with respect to the actual structure. The final test related to the protein 1A1Z is based on a starting structure that is perturbed by a molecular dynamics simulation. This presents a more challenging case since the structural alterations are distributed throughout the protein. The results of this exercise clearly show an improvement of the structural fitness to the RDC data from ~5 Hz error to ~0.03 (third entry of Table 2). The structural fitness measured as similarity to the actual structure is limited to 1.36Å, simply due to entrapment in a local minimum and is the result of utilizing a simplistic (Powell) minimization routine.

**Table 2:** Examination of the original 1A1Z structure and two different perturbations of 1A1Z, each in two alignment media, before and after refinement with XplorGUI

		** Before refinement**			** After refinement**	
**Starting structure**	**M1 (Hz)**	**M2 (Hz)**	**bb-rmsd(Å) to 1A1Z**	**M1 (Hz)**	**M2 (Hz)**	**bb-rmsd(Å) to 1A1Z**
1A1Z	1.3×10^-5^	3.9×10^-5^	0.00	0.0155	0.0153	0.22
1A1Z.PHI	2.38767	2.30944	1.99	0.0160	0.0155	0.23
1A1Z.NAMD	5.86019	4.57652	2.21	0.0352	0.0396	1.36

Further testing with experimental data from protein 1P7E revealed similar results listed in Table 3. For all three test cases, the fitness to the RDC data was improved for both alignment media following refinement (Table 3). Furthermore, comparisons between bb-rmsd values obtained before and after refinement for all structures reveal improvements in structural fitness (Table 3), although in some cases not as profoundly as those improvements noted in the case of experiments using 1A1Z. This observation can be explained by the impact of experimental noise and may also be due to the difference in the number of RDC sets per residue utilized. The availability of six RDC data per residue, in the case of 1A1Z, may simplify the RDC energy landscape of structural conformers and result in identification of the globally optimal structure by a Powell minimization. On the other hand, using only four RDCs per residue (as with 1P7E) may introduce a number of local minima, which may lead to entrapment of any gradient descent based search mechanisms (i.e. Powell minimization).

**Table 3:** Examination of the original 1P7E structure and two different perturbations of 1P7E, each in two alignment media, before and after refinement with XplorGUI

		** Before refinement**			** After refinement**	
**Starting structure**	**M1 (Hz)**	**M2 (Hz)**	**bb-rmsd (Å) to 1P7E**	**M1 (Hz)**	**M2 (Hz)**	**bb-rmsd (Å) to 1P7E**
1P7E	1.34176	0.98198	0.00	0.7563	0.5417	0.75
1P7E.PHI	5.61079	3.00242	2.18	0.7582	0.5468	1.55
1P7E.NAMD	13.0811	7.42607	2.15	0.7324	0.5786	1.50

### Structure validation

As described previously, a molecular dynamics simulation was created using the hTS protein in an effort to mimic localized structural distortions that may be found during protein structure analyses. Normally, examination of such instances with RDCs would indicate a poor structure (based on the global fitness of the structure to RDC data) and could be remedied by refinement of the entire structure. However, it is clear based on the nature of our simulation that structural variations are localized to only two regions (residues 100-135 and 174-204). Therefore, examining the localized fitness to RDC data would help to identify regions with high RDC violations and allow a typical refinement procedure to be applied to these regions only - sparing the remainder of the protein structure from any unnecessary distortions. This has motivated the Error-Analysis feature of REDCAT, which provides a localized sense of structural violation. Here we utilize the simulation exercise to demonstrate REDCAT’s ability to successfully isolate anomalous regions.

REDCAT analysis was used to quantify the structural fitness of the hTS protein to the simulated RDC data. The original structure of the hTS protein (PDB:1HVY) was loaded into REDCAT along with the averaged RDC data, which resulted from the simulation. A traditional assessment of the structural fitness to the RDC data produced an RDC-rmsd and Q-factor of 3.72Hz and 0.28, respectively for the first alignment medium and 3.39Hz and 0.28, respectively for the second alignment medium. These measures of fitness correspond to a structure that can benefit from additional refinement, but do not provide any information regarding the regions in need of further refinement.

**Figure 15:**
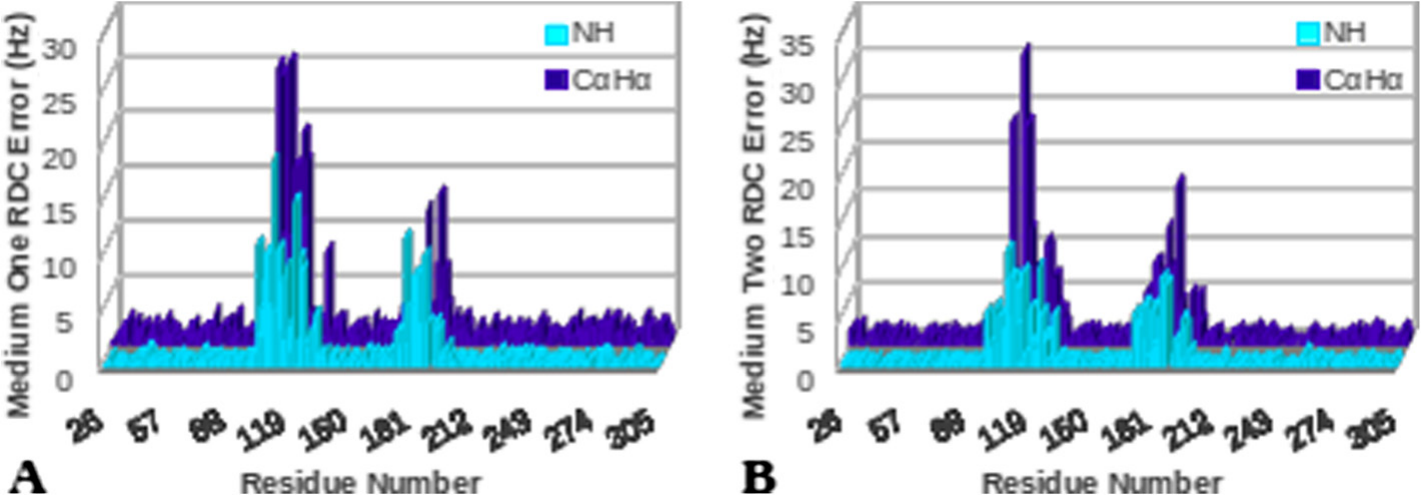
**Plot of errors generated by REDCAT analysis of NH and CαHα RDC vectors of the MDS.** For all residues in **(A)** medium 1 and **(B)** medium 2.

**Figure 16:**
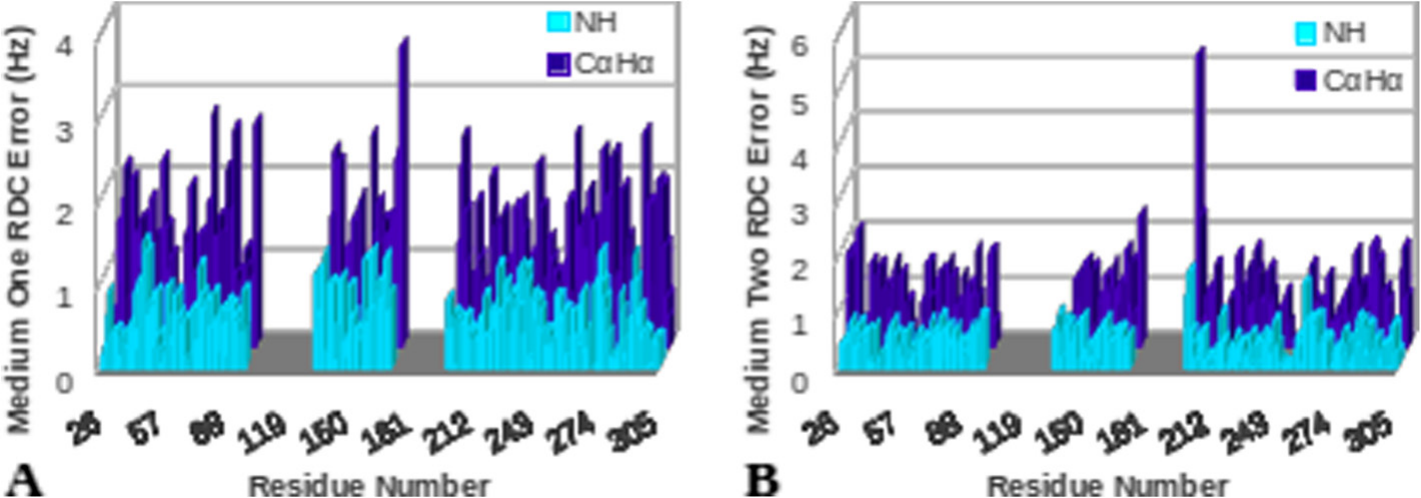
**Plot of errors generated by REDCAT analysis of NH and CαHα RDC vectors of the MDS.** After eliminating individual errors from the identified mobile domains (residues 101-135 and 175-202) in **(A)** medium 1 and **(B)** medium 2.

The results of Error-Analysis applied to the hTS data are shown in Figure 15. This figure illustrates the individual residual error for each of the backbone N-H and C_α_H_α_ vectors for media 1 and 2, respectively. Based on the results of these analyses it is evident that there are two regions (identified based on the severity of errors) with little agreement between the structure and the RDC data. REDCAT analysis identified these two regions as corresponding to residues 101 to 135 and residues 175 to 202, which correlate very well with the mobile domains within the molecular dynamics simulation of hTS. Additionally, independent identification of the same two regions by N-H and C_α_H_α_ RDCs from two alignment media provides additional reliability of the analysis. With this information established, subsequent analyses can be conducted to identify individual regions that exhibit acceptable agreement between the structure and corresponding RDC data. For example, results obtained after eliminating individual errors from the identified mobile domains (refer to Figure 16) confirm structural accuracy of the fixed regions, as evidenced by the lower RDC error values. Moreover, estimated order tensors from these regions are more meaningful for the purpose of structure refinement. Although this appears less evident in Figure 16B due to the higher RDC error value (5.319) of the C_α_H_α_ vector of residue 204, our finding is justified by the fact that this residue is after all contained in one of the mobile domains of the hTS simulation. Therefore, identification of the problematic regions in need of further treatment, combined with a more accurate estimate of order tensors, can lead to more efficient approaches to structure refinement.

## Conclusions

Study of structure and dynamics of biological macromolecules from RDC data is a very exciting prospect. However, the challenges and complications in analysis of RDC data often act as impediments in their use. The introduction of new features in REDCAT, combined with its interactions with VMD and Xplor-NIH, provide an environment for more extended analyses and utilization of RDCs. For example, the results of REDCAT’s Error-Analysis feature combined with visualization of RDC violations in the context of a related structure through interaction with VMD, can aid in determining the severity of violations and help in devising an effective strategy in refinement or treatment of those violations. Moreover, the reported interaction between the three software packages will easily assist in proper refinement of the initial structure by using more accurate estimates of order tensors, and effective isolation of the regions in need of refinement. Additionally, the visualization of the allowed orientation of the interacting vectors will provide a much more accurate perspective of the RDC violations; allowing a more meaningful inspection of the violations by observing the translated spatial violation, rather than just a numerical value. Finally, the newly introduced software engineering of REDCAT has been developed in the Object Oriented C++ environment. The culmination of these improvements will be of great interest to the community of researchers and developers, since it hides the complications of software development and permits an average user to engage without a full understanding of the programming details. In summary, our most recent enhancements to REDCAT serve to provide a more complete RDC analysis suite while also accommodating a more user-friendly experience.

## Availability and requirements

**Project name:** REDCAT

**Project home page:**http://ifestos.cse.sc.edu/

**Operating systems(s)**: Linux, Mac OSX

**Programming language:** C++, Java, Tcl/Tk

**Other requirements:** Tcl/Tk 8.5 or above, plotting software package gnuplot, and libtk-img library for Tcl/Tk environment

**License:** GNU Public License version 3 (GPLv3)

**Any restrictions to use by non-academics:** none

## Competing interests

The authors declare that they have no competing interests.

## Authors’ contributions

CS was involved in implementing the new features and improvements to REDCAT, performed the experiments and data analysis for structure refinement studies, and drafted the manuscript. SI performed the experiments and data analysis for studies involving structure validation, was involved in drafting the manuscript, and formatted the final manuscript. HV is responsible for the design and coordination of the study and helped draft the manuscript. All authors read and approved the final manuscript.
